# The low levels of eicosapentaenoic acid in rat brain phospholipids are maintained via multiple redundant mechanisms[S]

**DOI:** 10.1194/jlr.M038505

**Published:** 2013-09

**Authors:** Chuck T. Chen, Anthony F. Domenichiello, Marc-Olivier Trépanier, Zhen Liu, Mojgan Masoodi, Richard P. Bazinet

**Affiliations:** * Department of Nutritional Sciences, Faculty of Medicine, University of Toronto, Toronto, ON, Canada M5S 3E2; and; † Nestlé Institute of Health Sciences SA, Campus EPFL, Quartier de l'innovation, bâtiment G, 1015 Lausanne, Switzerland

**Keywords:** incorporation, turnover, kinetics, β-oxidation

## Abstract

Brain eicosapentaenoic acid (EPA) levels are 250- to 300-fold lower than docosahexaenoic acid (DHA), at least partly, because EPA is rapidly β-oxidized and lost from brain phospholipids. Therefore, we examined if β-oxidation was necessary for maintaining low EPA levels by inhibiting β-oxidation with methyl palmoxirate (MEP). Furthermore, because other metabolic differences between DHA and EPA may also contribute to their vastly different levels, this study aimed to quantify the incorporation and turnover of DHA and EPA into brain phospholipids. Fifteen-week-old rats were subjected to vehicle or MEP prior to a 5 min intravenous infusion of ^14^C-palmitate, ^14^C-DHA, or ^14^C-EPA. MEP reduced the radioactivity of brain aqueous fractions for ^14^C-palmitate-, ^14^C-EPA-, and ^14^C-DHA-infused rats by 74, 54, and 23%, respectively; while it increased the net rate of incorporation of plasma unesterified palmitate into choline glycerophospholipids and phosphatidylinositol and EPA into ethanolamine glycerophospholipids and phosphatidylserine. MEP also increased the synthesis of n-3 docosapentaenoic acid (n-3 DPA) from EPA. Moreover, the recycling of EPA into brain phospholipids was 154-fold lower than DHA. Therefore, the low levels of EPA in the brain are maintained by multiple redundant pathways including β-oxidation, decreased incorporation from plasma unesterified FA pool, elongation/desaturation to n-3 DPA, and lower recycling within brain phospholipids.

The brain has a unique FA composition with high levels of arachidonic acid (ARA) (20:4n-6) and docosahexaenoic acid (DHA) (22:6n-3), but low levels of other polyunsaturated fatty acids (PUFAs) such as eicosapentaenoic acid (EPA) (20:5n-3) (1–4). The maintenance of the unique PUFA composition in brain phospholipids is hypothesized to be the result of differences in uptake and/or metabolism upon FA entry into the brain (5, 6). Although brain EPA levels are low, several recent meta-analyses suggest that EPA is more effective than DHA for alleviating depressive symptoms (7–9). This poses a potential paradox as it is unclear how EPA, which is low within brain phospholipids, would be therapeutic (9).

In regard to receptor-mediated uptake, we established that the LDL receptor (10), VLDL receptor (11), or CD36 (12) are not necessary for maintaining brain PUFA concentrations. Thus, to examine the uptake of PUFA via simple passive diffusion, an in situ cerebral perfusion study was conducted. In addition to rapid passive diffusion, the rate of DHA and EPA uptake into the brain was similar (5, 13). Collectively, results from these studies suggested that the observed 250- to 300-fold difference between brain DHA and EPA concentrations is unlikely due to differences in uptake (14).

Previously, via in situ cerebral perfusion and in vivo intracerebroventricular infusion in rodents, we demonstrated that EPA was rapidly (5) and extensively (6) β-oxidized by the brain. Furthermore, we also observed that esterified EPA was rapidly lost from brain phospholipids to cellular metabolism at a rate of 14% per day (6) as compared with DHA (15), ARA, (16) and palmitate (6), all at 2% per day. However, there are other potential differences in metabolism between DHA and EPA that could contribute to their large difference in brain phospholipid levels. Therefore, one of the objectives of this study was to investigate further differences in the metabolism of DHA and EPA upon entry into the brain.

In addition to quantifying loss kinetics of EPA from brain phospholipids, the kinetics of EPA uptake, incorporation, and turnover can also be quantified via in vivo intravenous radiotracer infusion in rodents as described by Rapoport and others (17–19). The method of Rapoport allows for the calculation of brain kinetic parameters upon intravenous infusion of a high specific activity radiotracer at steady-state into a plasma pool available to the brain. Upon oral administration of a radiotracer, it appears in multiple pools and is not always at steady-state, making the calculation of brain kinetic parameters difficult, if not impossible (20). In this report, we quantified and compared four key kinetic parameters of palmitate, DHA, and EPA including k*, *J*_in_, *J*_FA_, and *F*_FA_. The incorporation coefficient (k*) describes the proportional uptake of radiolabeled FA from the plasma unesterified FA pool into stable brain phospholipids (17–19, 21–24). The *J*_in_ describes the net rate of plasma unesterified FA incorporation into brain phospholipids, whereas the *J*_FA_ describes the net rate of brain acyl-CoA incorporation into brain phospholipids (25). Lastly, the rate of turnover (*F*_FA_) describes the turnover of deesterified FA from phospholipids that are reesterified into brain phospholipid via Land's recycling (25).

Because the synthesis of PUFA within the brain is negligible relative to brain uptake (22, 23), the mathematical model can predict the relative contribution of plasma pools from which FAs enter the brain via comparison of the *J*_in_ to the net rate of loss from brain phospholipids, *J*_out_ (26). If the plasma unesterified PUFA pool is the major plasma contributor to brain phospholipid PUFA, then the *J*_in_ should approximate the *J*_out_. However, if the *J*_out_ exceeds the *J*_in_, then there may be additional plasma FA pools contributing to PUFA uptake into brain phospholipids. In contrast, if the *J*_in_ exceeds the *J*_out_, then it would suggest that either the measured kinetic parameters of PUFA are overestimations or modifications to the current model may be necessary.

Because β-oxidation appears to be a major contributor to the observed difference in brain PUFA concentrations (6), another objective of this study was to examine if mitochondrial FA β-oxidation is necessary for maintaining low levels of EPA in brain phospholipids by irreversibly inhibiting carnitine palmitoyltransferase (CPT)-Ia, the rate-limiting enzyme in mitochondrial FA β-oxidation that catalyzes the formation of acyl-carnitine from acyl-CoA, with methyl palmoxirate (MEP; methyl-2-tetradecylglycidate) (27–29). Although CPT-Ic is predominantly expressed in the brain (30), it is localized to the endoplasmic reticulum where it mediates food intake via endocannabinoids and ghrelin without modulating brain FA β-oxidation (31–35). Therefore, the inhibitory effects of MEP would likely be through CPT-Ia, the other isoform of CPT-I that is localized to the mitochondria in the brain (36). Previously, Freed et al. (27) reported that MEP treatment reduced the β-oxidation of ^14^C-palmitate and ^14^C-arachidonate. However, MEP treatment only increased the esterification of ^14^C-palmitate into brain total lipids and triacylglycerol. Overall, we found that MEP treatment led to significant increases in the *J*_in_ of EPA into ethanolamine glycerophospholipids (EtnGpl) and phosphatidylserine (PtdSer). Interestingly, there were also significant increases in n-3 docosapentaenoic acid (n-3 DPA) (22:5n-3) within choline glycerophospholipids (ChoGpl) and EtnGpl. Therefore, β-oxidation is involved, but not necessary for maintaining low EPA levels in brain phospholipids and elongation/desaturation of EPA into longer n-3 PUFA species may compensate for decreased β-oxidation in order to maintain low levels of EPA in brain phospholipids.

## MATERIALS AND METHODS

All procedures were performed in accordance with the policies set out by the Canadian Council on Animal Care and were approved by the Animal Ethics Committee at the University of Toronto. Male Sprague Dawley rats were purchased from Charles Rivers (Saint-Constant, QC, Canada) at 12 weeks of age and kept at the animal facility with an automated 12 h light-dark cycle and a constant temperature of 22°C for 3 weeks. The rats received ad libitum access to water and standard chow (Teklad 2018; Harlan, Madison, WI) which was composed of 54% of linoleate (18:2n-6), 5% of α-linolenate (18:3n-3), and <0.3% of longer chained PUFAs (20:2n-6, 20:3n-3, 20:4n-6, EPA, 22:4n-6, 22:5n-6, 22:5n-3, and DHA), as measured by gas chromatography-flame ionization detection (GC-FID).

### Radiotracer perfusate and MEP preparation

**Fig. 1.**
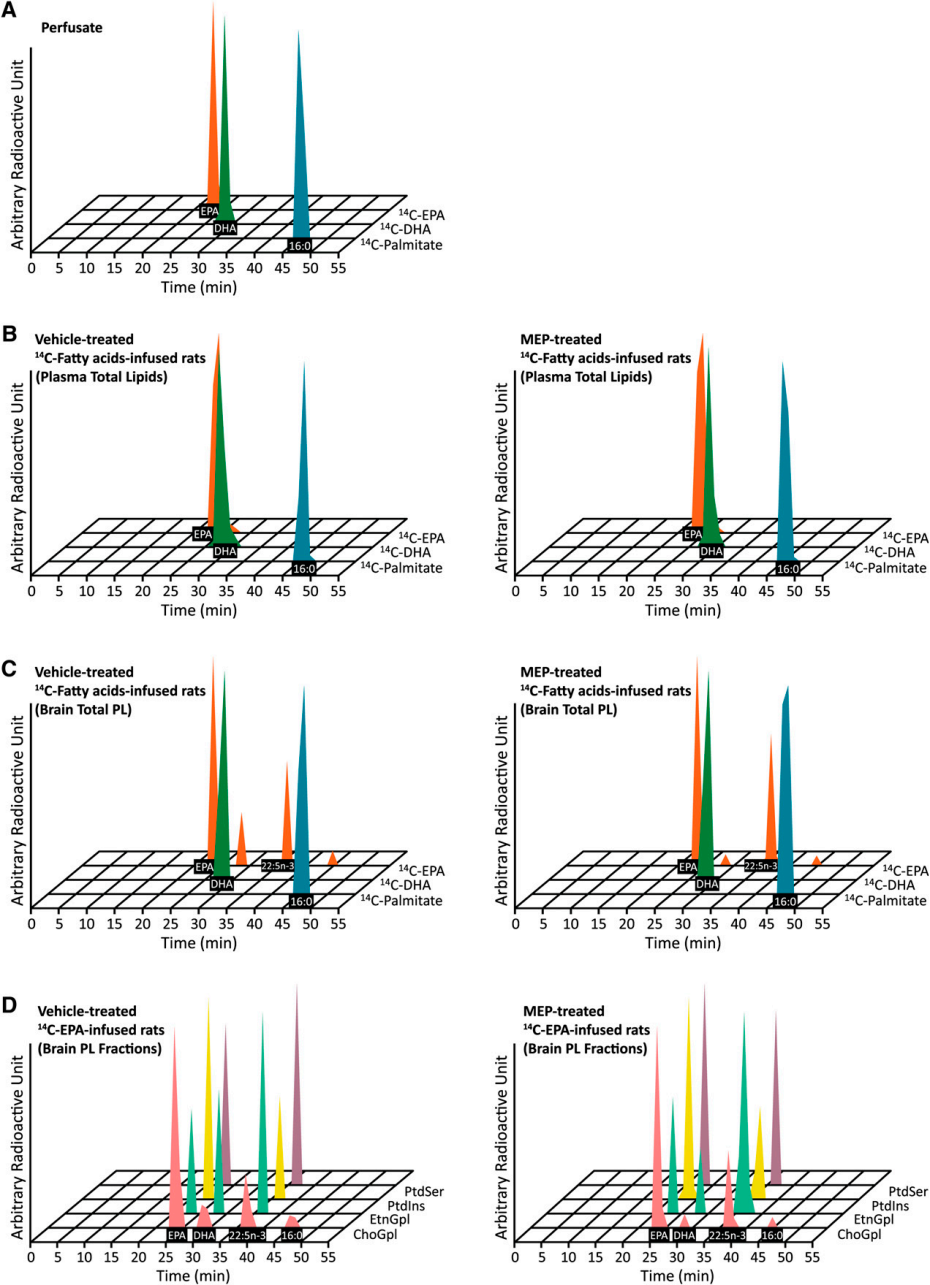
HPLC separation of brain radioactivity in (A) perfusates, (B) plasma total lipids of vehicle and MEP-treated rats, (C) brain total phospholipids (total PL) of vehicle and MEP-treated rats and (D) phospholipid classes of ^14^C-EPA-infused rats at 5 min post infusion. Peak identified was confirmed with authentic standards, GC-MS and GC-FID. No coelution with 16:0 (palmitate) and 22:5n-3 (n-3 DPA) was found. Percent composition of each identified radioactive FA was calculated and used to adjust the radioactivity of the total phospholipids.

Radiolabeled ^14^C-palmitate ([1-^14^C]palmitate, specific activity: 55 mCi/mmol; Moravek Biochemical Inc., Brea, CA), ^14^C-DHA ([1-^14^C]DHA, specific activity: 53 mCi/mmol; Moravek Biochemical Inc.), and ^14^C-EPA ([1-^14^C]EPA, specific activity: 54 mCi/mmol; Moravek Biochemical Inc.) were dissolved in 5 mM HEPES buffer (pH 7.4) containing 50 mg/ml FA-free BSA to a perfusate concentration of 66 μCi/ml. The purity of ^14^C-radiotracers was >99.9% as confirmed by high-performance liquid chromatography (HPLC) and liquid scintillation counting (LSC) (**Fig. 1A**). MEP (donated by Dr. S. I. Rapoport) was solubilized overnight in Tween 80 and diluted to 10 mg/ml in carboxymethylcellulose (0.1% in saline) (27).

### Surgery

Fifteen-week-old rats were anesthetized with isofluorane inhalation (3% induction, 1–2% maintenance). Rats were given a lower back subcutaneous injection of 5 mg/kg of ketoprofen (MERIAL Canada, Inc., Baie d'Urfé, QC, Canada). Polyethylene catheters (PE 50, Intramedic^™^; Becton Dickinson, Franklin Lakes, NJ) with silicone tubing [silicone tubing 0.020 inch inner diameter (I.D.) and 0.037 inch outer diameter(O.D.), VWR^®^; Mississauga, ON, Canada] filled with 0.9% saline were implanted into the right jugular vein. Surgery lasted for approximately 15 min. After surgery, all rats were singly housed to recover from anesthesia for at least 24 h with ad libitum access to food and water. Rats were not fasted for the radiotracer infusions.

### Free-living intravenous tracer infusion

Twenty-four hours postsurgery, a second catheter (iv catheter 24 gauge/0.75 inch, Angiocath^™^; Becton Dickinson) was implanted into the tail vein where rats received either vehicle or 10 mg/kg of MEP. Ten minutes post injection of vehicle or MEP, the tail vein catheter was connected to a syringe containing radiolabeled ^14^C-palmitate (n = 8), ^14^C-DHA (n = 6), or ^14^C-EPA (n = 7) attached to a computer-controlled variable speed pump (PHD 22/2000; Harvard Apparatus, Holliston, MA). The radiotracers were infused for 5 min via the tail vein catheter at a rate of 0.223(1 + e^−19.2t^) ml/min (*t* is infusion time in minutes) (37) which achieves a steady-state plasma radioactivity within 1 min (18, 38). Each rat received a dose of 76 μCi ^14^C-palmitate, 76 μCi ^14^C-DHA, or 78 μCi ^14^C-EPA. Thus a total of 1.39 μmol of palmitate, 1.42 μmol of DHA, or 1.44 μmol of EPA were infused over 5 min. During the 5 min infusion, blood samples were collected from the jugular vein at approximately 0, 0.25, 0.5, 0.75, 1.5, 3, 4, and 5 min while the unanesthetized rat was mobile in the infusion box with food and water. In a pilot study, we found that there were no significant differences in the plasma unesterified FA concentrations between carotid artery (palmitate, 163 ± 20 nmol/ml; EPA, 0.29 ± 0.017 nmol/ml, DHA, 0.63 ± 0.059 nmol/ml) and jugular vein (palmitate, 163 ± 22 nmol/ml; EPA, 0.31 ± 0.025 nmol/ml; DHA, 0.70 ± 0.058 nmol/ml). After 5 min, the rats were rapidly euthanized by head-focused high-energy microwave irradiation (13.5 kW for 1.6 s; Cober Electronics Inc., Stratford, CT). The radiotracer infusions continued until rats were euthanized. The brain was excised and cut sagittally. Both hemispheres were stored at −80°C for radioactive and biochemical analyses. Plasma was isolated from whole blood via microcentrifugation at 6,200 rpm (2,000 *g*) for 5 min and stored at −80°C.

### Lipid analysis

Total lipids from one brain hemisphere and from plasma were extracted by the method of Folch, Lees, and Sloane Stanley (39). Isolation of various neutral lipid and phospholipid classes from the total lipid extract was previously described by Chen, Liu, and Bazinet (6).

### Unesterified FA analysis

At 15 weeks of age, 16 rats were randomized to receive vehicle or 10 mg/kg MEP via tail vein catheter as described above. Fifteen minutes post injection, rats were euthanized by head-focused high energy microwave irradiation (13.5 kW for 1.6 s) and brains were excised and sagittally cut for unesterified FAs/lipid mediators and acyl-CoA analyses as described below. Brain hemispheres were homogenized in ethanol to yield a concentration of 100 mg tissue/ml. The unesterified FAs from 100 mg of brain tissue were isolated. The unesterified FA bands were collected and extracted twice from silica by hexane:isopropanol (3:2 by volume) with 5.5% water. Extracted unesterified FAs were dried down with nitrogen gas and added with 100 μl of freshly made pentafluorobenzylbromide (PFB) cocktail consisting of acetonitrile:N,N-diisopropylethylamine:PFB (1 ml:100 μl:10 μl by volume). The mixtures were shaken for 15 min and dried down with nitrogen gas. FA-PFB esters (FAPEs) were reconstituted in 100 μl hexane for GC-MS analysis as described below.

For measurements of unesterified EPA, brain samples were homogenized in ethanol on ice. An aliquot of 100 mg tissue was spiked with ARA-d8 (20 ng; Cayman Chemical, Ann Arbor, MI) and dried under nitrogen gas in reduced light conditions. Residues were dissolved in ethanol, acidified to pH 4 with 1 N HCl, and extracted three times with ethyl acetate. After washing to neutrality with water, the ethyl acetate fraction was dried under nitrogen and transferred to siliconized minivials for liquid chromatography-tandem mass spectrometry (LC-MS/MS) analysis as described below.

### Acyl-CoA analysis

Long chain acyl-CoA was extracted from the brains of vehicle- and MEP-treated rats as well as the brains of radiotracer-infused rats using a modified affinity chromatography method (40). Brains were homogenized by a probe sonicator in isopropanol:25 mM KH_2_PO_4_:acetonitrile (1:1:2 by volume) with 10 nmol of added internal standard, heptadecanoyl-CoA (17:0-CoA). Subsequently, protein was precipitated from the homogenate by the addition of saturated (NH_4_)_2_SO_4_. After centrifugation for 5 min at 3,000 rpm (2,000 *g*), supernatant containing acyl-CoA was extracted and diluted with a 1.25-fold volume of 25 mM KH_2_PO_4_. The diluted supernatant was then repeatedly passed through an oligonucleotide purification cartridge (ABI Masterpiece™, OPC^®^; Applied Biosystems, Foster City, CA) for three times at a rate of 5 ml/min. Afterwards, the cartridge was washed with 25 mM KH_2_PO_4_ and the bound acyl-CoA was eluted with isopropanol/1 mM glacial acetic acid (75:25 by volume). Acyl-CoA samples were reconstituted in elution buffer (100 μl) and stored at −80°C until HPLC analysis as described below.

### HPLC-ultraviolet photodiode array detection and LSC

FA methyl esters and radioactive acyl-CoA analysis were previously described by Chen, Liu, and Bazinet (6).

### LC-MS/MS

Acyl-CoA concentrations were detected using an Agilent 1200 Binary LC pump (Agilent Technologies, Wilmington, DE) equipped with a Zorbax SB-Phenyl column (3 × 50 mm, 3.5 μm spherical size; Chromatographic Specialties, Brockville, ON, Canada). The initial conditions of elution were set at 600 μl/min gradient system consisting of (A) 70% 10 mM ammonium acetate in water and (B) 30% 10 mM ammonium acetate:acetonitrile (10:90 by volume). The gradient started with 70% (A) and 30% (B) and maintained for 1 min, decreased to 30% (A) and 70% (B) over a 5 min period where it was maintained for 6 min before returning to 70% (A) and 30% (B) over a 6.2 min period and maintained for 10 min to complete the total run of 28.2 min. Mass spectrometry analyses were carried out on API4000 QTRAP (AB SCIEX, Concord, ON, Canada) quadruple-linear ion trap (QqLIT) mass spectrometers. The QTRAP analyses were conducted in positive ion mode under multiple reaction monitoring conditions. The turbospray temperature was set to 600°C, the curtain gas flow to 30 psi, and the ion spray voltage to 5,500 V. The collision energy, declustering potential, and collision cell exit potential were optimized and were set to 45, 42, 10 for palmitoyl-CoA; 45, 40, 7 for palmitoleoyl-CoA; 40, 40, 15 for heptadecanoyl-CoA; 47, 45, 10 for stearoyl-CoA; 47, 50, 10 for oleoyl-CoA and linoleoyl-CoA; 48, 40, 14 and 75, 40, 7 for α-linolenoyl-CoA; 43, 40, 15 for arachidonyl-CoA; 50, 40, 12 and 50, 40, 15 and 85, 40, 8 for EPA-CoA; and 40, 40, 15 for DHA-CoA. Peaks were identified and quantified by mass transitions, compound specific parameters, and standard curves of authentic acyl-CoA standards (Avanti, Alabaster, AL) (supplementary Fig. I). Concentrations were corrected for percent recovery of heptadecanoyl-CoA and expressed as nmol/g brain.

Brain unesterified EPA was detected using an Agilent HPLC 1200 equipped with a Zorbax SB-Phenyl column. HPLC solvent contained 4 μl/l propionic acid. The initial conditions of elution were set at 400 μl/min gradient system consisting of (A) water and (B) acetonitrile. The gradient started with 80% (A) and 20% (B) and maintained for 2 min, decreased to 75% (A) and 25% (B) for 0.5 min, then further decreased to 50% (A) and 50% (B) for 5 min, then to 45% (A) and 55% (B) for 6.2 min, and 100% (B) for 11 min. Mass spectrometry analyses were carried out on API5500 triple quadruple mass spectrometer (AB SCIEX, Concord, ON, Canada). The QTRAP analyses were conducted in electrospray ionization negative ion mode. The turbospray temperature was set to 500°C and the ion spray voltage to 4,500 V. The collision energy, declustering potential, and collision cell exit potential were optimized and were set to 15, 50, and 11, respectively. Concentration was quantified by comparing the deuterium-to-protium ratio of brain unesterified EPA with standard lines generated from authentic standards. Authentic standards in appropriate dilutions (0.002–2 ng) were prepared and analyzed simultaneously with brain samples. The lower limit of quantification (LLQ) was 0.002 ng (6.3 fmol of EPA).

### GC-FID

Brain FA concentrations from total and phospholipid classes were quantified as described by Chen, Liu, and Bazinet (6).

### GC-MS

FAPEs were identified with an Agilent 7890A gas chromatograph (Agilent Technologies) equipped with a SP-2380 (Supelco) fused silica column (Agilent Technologies; 30 m × 0.25 mm I.D. × 0.2 μm film thickness) and an Agilent 5975C quadruple mass spectrometry detector (Agilent Technologies). The sample was injected in split mode (10:1). The injection port temperature was set to 240°C and the ionization mode was set to negative chemical ionization using methane. FAPEs were eluted using a temperature program initially set at 150°C for 1 min, increase at 12°C/min to 270°C, and then at 40°C/min to 275°C for 3 min. The carrier helium gas was set to a constant flow of 1 ml/min. The LLQ for n-3 docosapentaenoate was 1 ng/20 mg brain. The LLQ for palmitate, palmitoleate, α-linolenate, and EPA was 5 ng/20 mg brain. The LLQ for ARA and linoleate was 10 ng/20 mg brain. The LLQ for oleate, stearate, and DHA was 20 ng/20 mg brain.

### Kinetics

Total and phospholipid class radioactivity were adjusted by the percentage of radiolabeled palmitate, DHA, and EPA as measured by HPLC and LSC for kinetic modeling. The model for in vivo kinetics of brain FAs in rats has been previously described (17, 18, 21, 23, 25, 37, 41).

The unidirectional incorporation coefficient, $k_{\text{i(palmitate, DHA, or EPA)}}^{*}$ (ml plasma/day/g brain), which represents the incorporation of plasma radiotracers into stable brain lipid pools *i*, was calculated as:

(Eq. 1a) $$k_{\text{i(palmitate, DHA, or EPA)}}^{*}=\frac{c_{\text{brain,}i\text{(palmitate, DHA, or EPA)}}^{*}(T)}{\int_{0}^{T}c_{\text{plasma(palmitate, DHA, or EPA)}}^{*}dt}$$

where $c_{\text{brain,}i\text{(palmitate, DHA, or EPA)}}^{\text{*}}$ is the radioactivity in *i* (nCi/g brain) from palmitate, DHA, or EPA at time *T*, and $c_{\text{plasma(palmitate, DHA, or EPA)}}^{*}$ is the plasma radioactivity (nCi/ml plasma) of ^14^C-palmitate-, ^14^C-DHA-, or ^14^C-EPA-infused rats.

Because elongation/desaturation products of ^14^C-EPA, n-3 DPA, and DHA were detected, we can determine their incorporation into stable lipid pools *i* with following adjustment to the equation:

(Eq. 1b) $$k_{\text{i(EPA}\to \text{n-3 DPA or DHA)}}^{*}=\frac{c_{\text{brain,}i\text{(n-3 DPA or DHA)}}^{*}(T)}{\int_{0}^{T}c_{\text{plasma(EPA)}}^{*}dt}$$

where $k_{\text{i(EPA}\to \text{n-3 DPA or DHA)}}^{*}$ is the conversion-incorporation coefficient and $c_{\text{brain,}i\text{(n-3 DPA or DHA)}}^{*}$ is the brain radioactivity of n-3 DPA or DHA as determined by HPLC and LSC.

Because the incorporation coefficient applies to both radiolabeled and nonradiolabeled FAs, we can determine the rate of incorporation of nonradiolabeled plasma unesterified FAs into stable brain lipid pools as unmetabolized FAs, $J_{\text{in,}i\text{(palmitate, DHA, or EPA)}}$, or as elongation/desaturation products $J_{\text{in,}i\text{(EPA}\to \text{n-3 DPA or DHA)}}$ (nmol/g brain/day).

(Eq. 2a) $$J_{\text{in,}i\text{(palmitate, DHA, or EPA)}}=k_{\text{i(palmitate, DHA, or EPA)}}^{*}c_{\text{plasma(palmitate, DHA, or EPA)}}$$

(Eq. 2b) $$J_{\text{in,}i\text{(EPA}\to \text{n-3 DPA or DHA)}}=k_{\text{i(EPA}\to \text{n-3 DPA or DHA)}}^{*}c_{\text{plasma(EPA)}}$$

where $c_{\text{plasma(palmitate, DHA ,or EPA)}}$ is the plasma unesterified FA concentration.

In addition to the rate of incorporation from plasma to stable brain lipid pools, we can also calculate the net rate of incorporation, $J_{\text{FA,}i\text{(palmitate, DHA, or EPA)}}$ (nmol/g brain/day) (18, 41), from the brain acyl-CoA pool to stable brain lipid pools via correction for the steady-state ratio of specific activity of the acyl-CoA pool over the specific activity of the radiotracer in plasma which is defined as the dilution factor, $\lambda_{\text{palmitate, DHA, or EPA}}$,

(Eq. 3) $$\lambda_{\text{palmitate, DHA, or EPA}}=\frac{c_{\text{brain(palmitate, DHA, or EPA-CoA)}}^{*}/c_{\text{brain(palmitate, DHA, or EPA-CoA)}}}{c_{\text{plasma(palmitate, DHA, or EPA)}}^{*}/c_{\text{plasma(palmitate, DHA, or EPA)}}}$$

where the numerator and denominator are the steady-state specific activities of brain acyl-CoA and plasma unesterified FAs, respectively. Because the infusion is 5 min, contributions of FA from de novo synthesis and esterified plasma FA are negligible (18, 20, 23, 41); thus only contributions from plasma unesterified FAs and acyl-CoA pools were considered in the calculation of $\lambda_{\text{palmitate, DHA, or EPA}}$.

$J_{\text{FA,}i\text{(palmitate, DHA ,or EPA)}}$ of nonradiolabeled FAs and EPA elongation/desaturation products from acyl-CoA pools into stable brain lipid pools *i* are calculated as followed:

(Eq. 4a) $$J_{\text{FA,}i\text{(palmitate, DHA, or EPA)}}=\frac{J_{\text{in,}i\text{(palmitate, DHA, or EPA)}}}{\lambda_{\text{palmitate, DHA, or EPA}}}$$

(Eq. 4b) $$J_{\text{FA,}i\text{(EPA}\to \text{n-3 DPA or DHA)}}=\frac{J_{\text{in,}i\text{(EPA}\to \text{n-3 DPA or DHA)}}}{\lambda_{\text{EPA}}}$$

The rate of turnover, $F_{\text{FA,}i\text{(palmitate, DHA, or EPA)}}$ (%/day), and half-life, t_1/2_ (day) (18, 41), within stable brain lipid pools *i* as unmetabolized FAs or EPA elongation/desaturation products are quantified as,

(Eq. 5a) $$F_{\text{FA,}i\text{(palmitate, DHA, or EPA)}}=\frac{J_{\text{FA,}i\text{(palmitate, DHA, or EPA)}}}{c_{\text{brain,}i\text{(palmitate, DHA, or EPA)}}}$$

(Eq. 5b) $$F_{\text{FA,}i\text{(EPA}\to \text{n-3 DPA or DHA)}}=\frac{J_{\text{FA,}i\text{(EPA}\to \text{n-3 DPA or DHA)}}}{c_{\text{brain,}i\text{(n-3 DPA or DHA)}}}$$

where $c_{\text{brain,}i\text{(palmitate, DHA, or EPA)}}$ is the brain FA concentrations of stable brain lipid pools *i*.

(Eq. 6a) $${\text{t}}_{1/2}=\frac{0.693}{F_{\text{FA,}i\text{(palmitate, DHA, or EPA)}}}$$

(Eq. 6b) $${\text{t}}_{1/2}=\frac{0.693}{F_{\text{FA,}i\text{(EPA}\to \text{n-3 DPA or DHA)}}}$$

### Statistics

Concentrations and rates are expressed as mean ± SD. HPLC profiles were analyzed as pooled samples and do not have SD. Statistical comparisons of kinetic parameters between ^14^C-DHA and ^14^C-EPA infusions were performed, a priori, using two-tailed *t*-test. Differences between ^14^C-DHA and ^14^C-EPA infusions upon vehicle administration were statistically significant at ^#^*P* < 0.05, ^##^*P* < 0.01, and ^###^*P* < 0.001. Comparisons were not performed with ^14^C-palmitate which served as positive control to confirm the activity of MEP (27). Statistical comparisons of FA and acyl-CoA concentrations, radioactivity, and kinetic parameters between vehicle- and MEP-treated rats were performed using two-tailed *t*-test. All data had passed the normality and equal variance test (SigmaStats 3.5). Differences between vehicle- and MEP-treated rats were statistically significant at *P < 0.05, **P < 0.01, and ***P < 0.001.

## RESULTS

### Brain FA and acyl-CoA concentrations

Upon MEP treatment, there were selective increases in brain unesterified FAs (**Table 1**). There were significant 2.5-, 1.4-, 1.5-, and 1.3-fold increases in unesterified palmitate, linoleate (18:2n-6), α-linolenate (18:3n-3), and EPA, respectively. In contrast, ARA (20:4n-6) and DHA were unaffected by MEP.

**TABLE 1.** Brain FA concentrations of unesterified FAs, acyl-CoA, and total phospholipids

	Brain Unesterified FAs		Brain Acyl-CoA		Brain Total PL	
	Vehicle	MEP	Vehicle	MEP	Vehicle	MEP
16:0	22 ± 16	54 ± 24**	6.2 ± 0.33	6.4 ± 0.59	24,559 ± 2030	23,932 ± 725
16:1n-7	0.36 ± 0.13	0.49 ± 0.1*	1.2 ± 0.11	1.3 ± 0.2	376 ± 65	327 ± 48
18:0	38 ± 10	79 ± 31**	6.1 ± 0.39	5.4 ± 0.68*	22,241 ± 2,610	21,349 ± 561
18:1n-9	6.6 ± 2.7	8.2 ± 1.2	8.3 ± 0.54	7.8 ± 0.74	21,384 ± 3,400	20,370 ± 960
18:2n-6	0.91 ± 0.36	1.3 ± 0.31*	0.47 ± 0.048	0.63 ± 0.11**	770 ± 49	743 ± 52
18:3n-3	0.074 ± 0.036	0.11 ± 0.061*	0.022 ± 0.0039	0.043 ± 0.016**	27 ± 6.5	24 ± 2.7
20:4n-6	3.8 ± 0.95	4.3 ± 1.8	0.90 ± 0.065	0.83 ± 0.01	7,921 ± 791	7,797 ± 318
20:5n-3^+^	0.019 ± 0.0017	0.025 ± 0.0051**	0.019 ± 0.0012	0.016 ± 0.0041*	225 ± 37	202 ± 29
22:5n-3	0.13 ± 0.02	0.13 ± 0.061	ND	ND	17 ± 3	16 ± 2
22:6n-3	6.0 ± 2	7.0 ± 1.4	0.68 ± 0.028	0.50 ± 0.093***	8,789 ± 830	8,729 ± 380

Unesterified FAs (n = 8 per treatment), acyl-CoA (n = 8 per treatment), and total phospholipids (total PL) (n = 10–11 per treatment). Data are mean ± SD and are expressed in nmol/g brain. Unesterified FA concentrations were quantified by GC-MS with exception of EPA^+^ which was determined by LC-MS/MS. Total phospholipid FA concentrations were quantified by GC-FID. Acyl-CoA concentrations were quantified by LC-MS/MS. *P* values indicated significantly different from vehicle-treated rats; *P < 0.05, **P < 0.01, ***P < 0.001. Brain 22:5n-3 (n-3 DPA)-CoA was not determined (ND).

MEP had no effect on the concentration of palmitoyl-CoA but reduced the concentration of stearoyl-CoA (Table 1). MEP increased linoleoyl-CoA and α-linolenoyl-CoA and decreased EPA-CoA. Interestingly, while MEP had no effect on unesterified DHA, it significantly reduced DHA-CoA in the brain.

Acute administration of MEP was insufficient to significantly alter total phospholipid FA concentrations (Table 1). There were no significant differences between the brain FA concentrations of all radiotracer-infused rats in each treatment; therefore, data were pooled for comparison between vehicle and MEP treatments. In accordance with previous reports, palmitate, stearate, and oleate were the major constituents of brain phospholipids consisting of 25, 22, and 21% of the total FAs, respectively. Similarly, the major PUFA species in brain phospholipids were ARA and DHA, which accounted for 8% and 9% of total FAs, respectively. Lastly, the level of EPA in brain total phospholipids was relatively low, as compared with DHA, at 0.2% of total FAs which corresponded to 225 ± 12 nmol/g brain in vehicle-treated rats and 202 ± 8.6 nmol/g brain in MEP-treated rats.

After fractionation into the four major phospholipid classes, there was no significant effect of MEP on the FA compositions of individual phospholipid classes with the exceptions of a 9% reduction in linoleate from EtnGpl and an 8% reduction in ARA from PtdSer upon MEP treatment (**Table 2**). After adjustments for pool size, palmitate was primarily esterified to ChoGpl and as the major component in ChoGpl, it accounted for 48% of total ChoGpl FAs (Table 2). As for DHA, it was primarily esterified to EtnGpl and PtdSer which accounted for 17% of total FAs in both EtnGpl and PtdSer (Table 2). As for EPA, albeit, the esterification of EPA to phosphatidylinositol (PtdIns) was the lowest among four major phospholipid classes, when pool size is considered, 0.5% of total PtdIns FAs were EPA as compared with 0.1, 0.2, and 0.4% of total ChoGpl, EtnGpl, and PtdSer FAs, respectively.

**TABLE 2.** Brain FA concentrations of four major phospholipid classes

	ChoGpl		EtnGpl		PtdIns		PtdSer	
	Vehicle	MEP	Vehicle	MEP	Vehicle	MEP	Vehicle	MEP
16:0	23,818 ± 1,531	23,762 ± 492	2,903 ± 242	2,786 ± 107	807 ± 199	746 ± 67	367 ± 89	333 ± 23
16:1n-7	146 ± 14	145 ± 11	148 ± 62	143 ± 45	12 ± 3	11 ± 1.3	6.0 ± 1.2	5.8 ± 1.1
18:0	6,597 ± 646	6,403 ± 150	6,590 ± 257	6,492 ± 213	1,933 ± 355	1,811 ± 138	6,441 ± 704	6,104 ± 414
18:1n-9	10,893 ± 1,098	10,705 ± 373	8,833 ± 973	8,235 ± 507	864 ± 199	813 ± 72	3,165 ± 528	2,899 ± 278
18:2n-6	464 ± 32	452 ± 36	182 ± 10	166 ± 11**	44 ± 10	41 ± 5.2	26 ± 6	24 ± 5.4
18:3n-3	11 ± 1.3	10 ± 0.83	14 ± 3.1	13 ± 1.5	1.2 ± 0.32	1.2 ± 0.15	2.9 ± 0.46	2.7 ± 0.46
20:4n-6	2,242 ± 169	2,255 ± 70	4,302 ± 322	4,089 ± 154	1,330 ± 226	1,279 ± 126	449 ± 46	411 ± 32*
20:5n-3	61 ± 9	60 ± 8.7	74 ± 16	65 ± 11	26 ± 12	24 ± 6.2	57 ± 17	50 ± 9.4
22:5n-3	3.7 ± 0.65	3.5 ± 0.37	8.6 ± 0.73	8.7 ± 1	0.81 ± 0.27	0.69 ± 0.13	5.8 ± 0.84	5.7 ± 0.5
22:6n-3	1,351 ± 100	1,387 ± 85	5,812 ± 485	5,448 ± 356	196 ± 49	185 ± 40	2,357 ± 161	2,331 ± 105

Data are mean ± SD and are expressed in nmol/g brain (n = 10–11 per treatment). FA concentrations were quantified by GC-FID. *P* values indicated significantly different from vehicle-treated rats; *P < 0.05, **P < 0.01.

### Identification of radioactivity in total and phospholipid fractions

After a 5 min infusion of radiolabeled ^14^C-palmitate, the only radioactive peak in plasma total lipids and brain total phospholipids of the vehicle- and MEP-treated rats was palmitate (Fig. 1B, C). Similarly, upon ^14^C-DHA infusion, the only radioactive peak in plasma total lipids and brain total phospholipids of vehicle- and MEP-treated rats was DHA (Fig. 1B, C). Upon ^14^C-EPA infusion, while plasma total lipids of the vehicle- and MEP-treated rats only contained radioactive EPA peak (Fig. 1B), brain total phospholipids from the vehicle- and MEP-treated rats all contained radiolabeled EPA, DHA, n-3 DPA, and palmitate (Fig. 1C). However, we observed higher n-3 DPA and lower DHA in the MEP-treated rats. The major radioactive peak in brain total phospholipids of the vehicle- and MEP-treated rats was EPA, accounting for 62 and 56% of total radiolabeled FAs, respectively; whereas the minor peak was palmitate, accounting for 4 and 2% of brain total radiolabeled FAs in the vehicle- and MEP-treated rats, respectively. In the vehicle-treated rats, 24 and 10% of brain total radiolabeled FAs corresponded to n-3 DPA and DHA, respectively, elongation and desaturation products of EPA; while in the MEP-treated rats, the composition of radiolabeled n-3 DPA and DHA was 38 and 3% of brain total radiolabeled FAs, respectively.

Because we detected elongation and desaturation products in the ^14^C-EPA-infused rats, the percent composition of radiolabeled FAs in each major phospholipid class was also measured (Fig. 1D). The radiolabeled FA composition of ChoGpl from the vehicle-treated rats was 65% EPA, 11% DHA, 18% n-3 DPA, and 6% palmitate; whereas the composition from the MEP-treated rats was 66% EPA, 4% DHA, 26% n-3 DPA, and 3% palmitate. In contrast to ChoGpl, only three of the four radiolabeled FAs were detected in EtnGpl. From EtnGpl of the vehicle-treated rats, the radiolabeled FA composition was 24% EPA, 29% DHA, and 47% n-3 DPA; while in the MEP treated rats, the composition was 25% EPA, 14% DHA, and 61% n-3 DPA. In PtdIns and PtdSer fractions, there were only two detectable radiolabeled peaks, EPA and n-3 DPA. In PtdIns of the vehicle- and MEP-treated rats, EPA accounted for the majority of brain radiolabeled FAs at 66 and 65%, respectively. However, in PtdSer, there was more radiolabeled EPA (54% of total radiolabeled FAs) in the MEP-treated rats as opposed to the vehicle-treated rats (44% of radiolabeled FAs). Lastly, there was no esterification of ^14^C-EPA into ceramide phosphocholine after 5 min of infusion (data not shown).

### Radioactivity in brain aqueous and lipid fractions

**Fig. 2.**
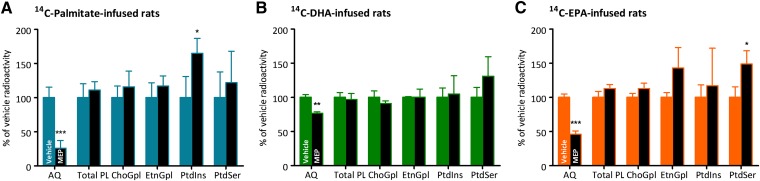
Radioactivity of the aqueous (AQ) and organic fractions including total phospholipids (total PL) and four major phospholipid fractions (n = 3–4) upon HPLC adjustment in (A) ^14^C-palmitate-infused, (B) ^14^C-DHA-infused and (C) ^14^C-EPA-infused rats. Data are mean ± SD and are expressed in nCi/g brain. Fractions were isolated by TLC and radioactivity counted by LSC. *P* values indicated significantly different from vehicle-treated rats; *P < 0.05, **P < 0.01, ***P < 0.001.

**Fig. 3.**
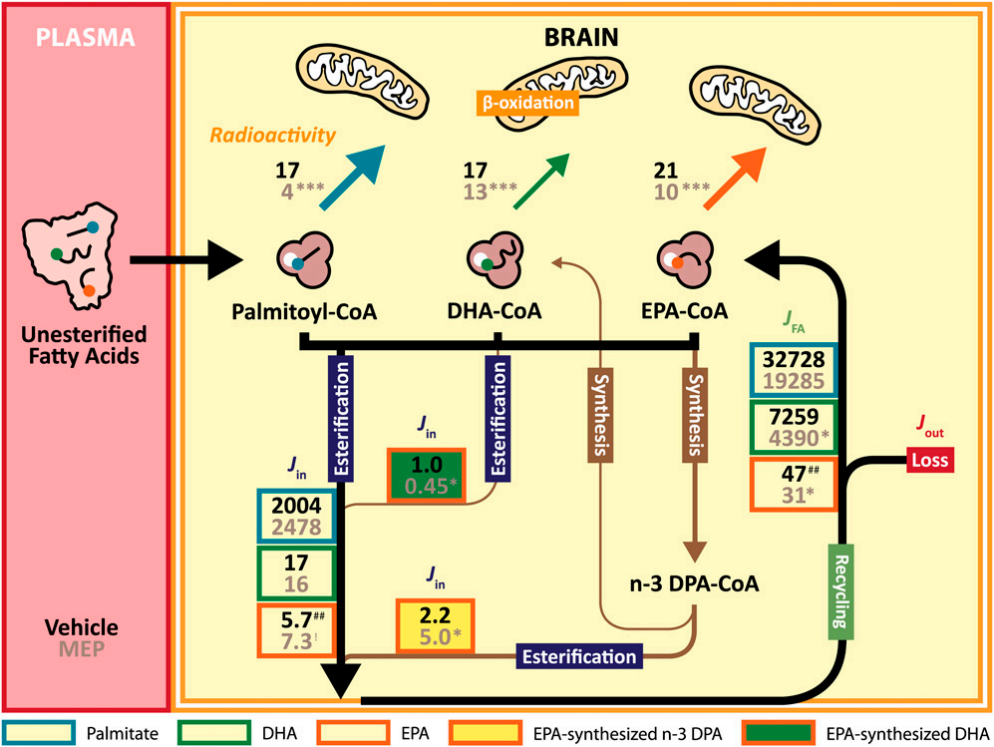
Kinetic summary of palmitate, DHA, and EPA in brain total phospholipids for vehicle (black) and MEP-treated (gray) rats. Kinetic rates (*J*_in_ and *J*_FA_) are nmol/g brain/day and radioactivity is nCi/g brain. *P* values indicated significantly different from vehicle-treated rats; *P < 0.05, ***P < 0.001, ^!^*P* = 0.06. *P* values indicating significant differences between ^14^C-DHA-infused and ^14^C-EPA-infused rats are ^##^*P* < 0.01.

MEP significantly decreased radioactivity in the brain aqueous fraction (marker of β-oxidation) for all radiotracers (**Fig. 2**). Upon vehicle injections, the radioactivity of aqueous fractions between radiotracer-infused rats were similar (^14^C-palmitate, 17 nCi/g brain; ^14^C-DHA, 17 nCi/g brain; ^14^C-EPA, 21 nCi/g brain) (**Fig. 3**). However, post MEP treatment, radioactivity in the brain aqueous fraction of the ^14^C-palmitate-infused rats was significantly reduced by 74%, whereas in the ^14^C-EPA-infused rats and the ^14^C-DHA-infused rats the radioactivities of the brain aqueous fractions were reduced by 54 and 23%, respectively.

In ^14^C-palmitate-infused rats, MEP had no significant effect on esterification into brain total phospholipids (Fig. 2). However, there was a significant 65% increase in radioactivity of PtdIns in MEP-treated rats; while no significant differences were observed in ChoGpl, EtnGpl, and PtdSer between the brains of the vehicle- and MEP-treated rats (Fig. 2). In ^14^C-DHA-infused rats, MEP had no significant effect on brain total phospholipids or any individual phospholipid classes (Fig. 2). Finally, upon ^14^C-EPA infusion, MEP significantly increased total radioactivity in brain total phospholipids (vehicle, 17 ± 0.9 nCi/g brain; MEP, 22 ± 0.6 nCi/g brain; *P* < 0.05) (data not shown). However, upon adjusting the radioactivity for percent composition of ^14^C-EPA, we observed no significant effect of MEP treatment on esterification of ^14^C-EPA into brain total phospholipids (*P* = 0.06), but esterification into PtdSer was increased upon MEP treatment (Fig. 2). It is possible with a larger sample size that we would have observed a significant effect of MEP on ^14^C-EPA incorporation into total phospholipids and this result should be interpreted with caution. The increase in total radioactivity upon MEP treatment was largely due to a significant increase in the esterification of radiolabeled n-3 DPA into brain total phospholipids (vehicle, 4.1 ± 0.2 nCi/g brain; MEP, 8.3 ± 0.2 nCi/g brain; *P* < 0.001) (data not shown). There was also a significant 49% increase in radioactivity of PtdSer with MEP treatment; while other phospholipid classes were unaffected by MEP (Fig. 2).

Furthermore, to account for the amount of infused radiotracer in the plasma available to the brain, incorporation coefficients [*k*_i_* (Eq. 1)] were determined. There was a significant increase in *k*_i_* of ^14^C-palmitate into ChoGpl and PtdIns upon MEP treatment (**Table 3**). Upon ^14^C-DHA infusion, there was no effect of MEP on *k*_i_* (Table 3). In ^14^C-EPA-infused rats, there was no effect of MEP on *k*_i_* of brain total phospholipids. However, there were significant increases in *k*_i_* for ^14^C-EPA into EtnGpl in addition to PtdSer (Table 3).

**TABLE 3.** Kinetic parameters in rat brain total phospholipids and four major phospholipid classes

	*k*_i_* (ml/day/g)		*J*_in_ (nmol/g/day)		*J*_FA_ (nmol/g/day)		*F*_FA_ (%/day)		t_1/2_ (day)	
	Vehicle	MEP	Vehicle	MEP	Vehicle	MEP	Vehicle	MEP	Vehicle	MEP
Palmitate										
Total PL	12 ± 2.9	15 ± 2.8	2,004 ± 468	2,478 ± 458	32,728 ± 17,692	19,285 ± 6,630	127 ± 57	80 ± 30	0.64 ± 0.29	0.95 ± 0.32
ChoGpl	8.0 ± 1.3	10 ± 0.82*	1,297 ± 214	1,649 ± 134*	20,713 ± 9,692	12,720 ± 3,120	84 ± 33	53 ± 13	0.92 ± 0.33	1.4 ± 0.38
EtnGpl	1.9 ± 0.35	2.4 ± 0.4	303 ± 56	393 ± 65	4,926 ± 2,623	3,062 ± 1,011	161 ± 66	110 ± 37	0.49 ± 0.18	0.69 ± 0.24
PtdIns	1.0 ± 0.23	1.9 ± 0.61*	161 ± 38	310 ± 100*	2,679 ± 1,655	2,443 ± 1,153	292 ± 80	311 ± 135	0.25 ± 0.073	0.25 ± 0.098
PtdSer	0.49 ± 0.28	0.65 ± 0.33	80 ± 46	105 ± 54	1,253 ± 792	849 ± 562	356 ± 259	261 ± 194	0.26 ± 0.13	0.39 ± 0.24
DHA										
Total PL	28 ± 2.9	25 ± 1.9	17 ± 1.8	16 ± 1.2	7,259 ± 958	4,390 ± 988*	75 ± 7.7	51 ± 10*	0.92 ± 0.089	1.4 ± 0.26
ChoGpl	8.2 ± 0.99	6.9 ± 0.61	5.2 ± 0.62	4.3 ± 0.38	2,146 ± 225	1,234 ± 344*	150 ± 14	90 ± 20*	0.47 ± 0.044	0.79 ± 0.16
EtnGpl	13 ± 2.2	12 ± 1.9	7.9 ± 1.4	7.2 ± 1.2	3,233 ± 184	2,030 ± 492*	55 ± 5.4	37 ± 9.6	1.3 ± 0.13	1.9 ± 0.47
PtdIns	2.8 ± 0.12	2.7 ± 0.48	1.8 ± 0.078	1.7 ± 0.3	688 ± 144	477 ± 106	458 ± 156	308 ± 105	0.16 ± 0.052	0.24 ± 0.081
PtdSer	0.90 ± 0.084	1.1 ± 0.18	0.56 ± 0.053	0.68 ± 0.11	237 ± 50	198 ± 79	9.2 ± 2.1	8.4 ± 3.1	7.6 ± 1.5	9.1 ± 3.7
EPA										
Total PL	20 ± 4	26 ± 2.4	5.7 ± 1.1^##^	7.3 ± 0.7^!^	47 ± 5.7^##^	31 ± 4.8*	21 ± 2.9^##^	17 ± 2.3	3.3 ± 0.45^#^	4.2 ± 0.58
ChoGpl	9.9 ± 1.9	13 ± 1.1^#^	2.8 ± 0.55^##^	3.7 ± 0.31^!^	23 ± 3.3^##^	16 ± 2.3*	46 ± 6.9^##^	28 ± 4.5*	1.5 ± 0.22^#^	2.5 ± 0.47*
EtnGpl	2.7 ± 0.66^#^	4.3 ± 0.61*	0.78 ± 0.19^#^	1.2 ± 0.17*	6.4 ± 1.3^###^	5.4 ± 1.4	8.4 ± 0.81^##^	9.5 ± 3.3	8.3 ± 0.8^##^	8.4 ± 4.2
PtdIns	1.8 ± 0.3^#^	2.5 ± 1.4	0.52 ± 0.087^###^	0.73 ± 0.41	4.5 ± 0.2^#^	3.0 ± 1.4	24 ± 1.7^#^	17 ± 6.6	2.9 ± 0.2^###^	4.4 ± 1.4
PtdSer	0.60 ± 0.19	1.0 ± 0.2*	0.17 ± 0.055^###^	0.29 ± 0.057*	1.4 ± 0.36^#^	1.2 ± 0.13	3.1 ± 0.56^#^	3.1 ± 0.43	23 ± 4.4^#^	23 ± 2.9
EPA→n-3 DPA										
Total PL	7.6 ± 1.5	17 ± 1.7***	2.2 ± 0.44	5.0 ± 0.49**	18 ± 2.2	21 ± 3.3	123 ± 34	149 ± 35	0.59 ± 0.15	0.48 ± 0.12
ChoGpl	2.7 ± 0.53	5.0 ± 0.43**	0.79 ± 0.15	1.4 ± 0.12**	6.5 ± 0.92	6.2 ± 0.91	192 ± 25	178 ± 41	0.36 ± 0.047	0.40 ± 0.09
EtnGpl	5.2 ± 1.3	11 ± 1.5**	1.5 ± 0.36	3.0 ± 0.43**	12 ± 2.5	13 ± 3.5	147 ± 30	153 ± 44	0.49 ± 0.1	0.48 ± 0.13
PtdIns	0.93 ± 0.16	1.4 ± 0.78	0.27 ± 0.045	0.39 ± 0.22	2.3 ± 0.1	1.6 ± 0.77	336 ± 62	260 ± 113	0.21 ± 0.041	0.31 ± 0.15
PtdSer	0.75 ± 0.24	0.88 ± 0.17	0.21 ± 0.068	0.25 ± 0.05	1.8 ± 0.45	1.1 ± 0.11	31 ± 11	20 ± 3.6	2.2 ± 0.6	3.6 ± 0.61*
EPA→DHA										
Total PL	3.4 ± 0.67	1.6 ± 0.15*	1.0 ± 0.19	0.45 ± 0.044*	7.9 ± 0.97	1.9 ± 0.3*	0.091 ± 0.0089	0.022 ± 0.0035*	768 ± 72	3,195 ± 583*
ChoGpl	1.6 ± 0.31	0.80 ± 0.069**	0.46 ± 0.089	0.23 ± 0.02**	3.8 ± 0.54	0.98 ± 0.14**	0.30 ± 0.035	0.070 ± 0.01**	233 ± 28	1,010 ± 146**
EtnGpl	3.2 ± 0.78	2.5 ± 0.35	0.92 ± 0.22	0.71 ± 0.1	7.6 ± 1.5	3.1 ± 0.81*	0.13 ± 0.023	0.054 ± 0.017*	556 ± 93	1,370 ± 415*
PtdIns	ND	ND	ND	ND	ND	ND	ND	ND	ND	ND
PtdSer	ND	ND	ND	ND	ND	ND	ND	ND	ND	ND

Data are mean ± SD. *P* values indicated significantly different from vehicle-treated rats; *P < 0.05, **P < 0.01, ***P < 0.001, ^!^*P* = 0.06. *P* values indicating significant differences between ^14^C-DHA-infused and ^14^C-EPA-infused rats upon vehicle injection are ^#^*P* < 0.05, ^##^*P* < 0.01, ^###^*P* < 0.001. Total PL, total phospholipids; ND, not detectable.

### Net rates of incorporation of palmitate, DHA, and EPA from plasma unesterified FA and brain acyl-CoA pools into brain phospholipids

The *J*_in_ (Eq. 2) of palmitate was significantly higher in ChoGpl and PtdIns upon MEP treatment (Table 3). There were no significant effects of MEP on the *J*_in_ of DHA into brain phospholipids (Table 3). Upon MEP treatment, the *J*_in_ for EPA was significantly increased in EtnGpl and PtdSer (Table 3). Additionally, there were significant increases in the *J*_in_ of EPA-synthesized n-3 DPA into brain total phospholipids, ChoGpl and EtnGpl of the MEP-treated rats (Table 3). However, this was accompanied by significant decreases in the *J*_in_ of EPA-synthesized DHA into brain total phospholipids and ChoGpl of the MEP-treated rats (Table 3).

MEP did not significantly affect the *J*_FA_ (Eq. 4) of palmitoyl-CoA into brain phospholipids (Table 3). In ^14^C-DHA-infused rats, MEP significantly reduced the *J*_FA_ of DHA-CoA in brain total phospholipids, ChoGpl and EtnGpl (Table 3). Similarly, in ^14^C-EPA-infused rats, there were significant reductions in the *J*_FA_ of EPA-CoA in brain total phospholipids and ChoGpl with MEP treatment (Table 3). Additionally, MEP did not affect the *J*_FA_ of EPA-synthesized n-3 DPA-CoA into brain phospholipids (Table 3). However, in accordance with MEP's effect on the *J*_FA_ of DHA-CoA, MEP also significantly reduced the *J*_FA_ of EPA-synthesized DHA-CoA into brain total phospholipids, ChoGpl and EtnGpl (Table 3).

In comparing *k*_i_* between DHA and EPA, there was no significant difference between the *k*_i_* (Table 3). However, the *J*_in_ of DHA into brain total phospholipids was 3-fold higher than EPA upon vehicle-injection (Table 3). In addition, the *J*_FA_ of DHA-CoA into total phospholipids was 156-fold higher as compared with EPA-CoA upon vehicle injection (Table 3).

### Rate of turnover of palmitate, DHA, and EPA in brain phospholipids

While MEP did not affect the rate of turnover, *F*_FA_ (Eq. 5), of palmitate and EPA in brain total phospholipids, MEP significantly reduced the *F*_FA_ of DHA in brain total phospholipids by 24% per day (Table 3). When individual phospholipid classes were analyzed, MEP did not significantly alter the *F*_FA_ of palmitate in any phospholipid classes (Table 3). However, MEP significantly reduced the *F*_FA_ of EPA and DHA in ChoGpl (Table 3). Similar to MEP's effect on the *F*_FA_ for DHA, MEP also significantly reduced the *F*_FA_ of EPA-synthesized DHA to brain total phospholipids, ChoGpl and EtnGpl; whereas there was no effect on EPA-synthesized n-3 DPA (Table 3). The *F*_FA_ of DHA into brain total phospholipids was 4-fold higher as compared with EPA upon vehicle injection (Table 3).

In regard to half-life, t_1/2_ (Eq. 6), MEP did not significantly affect the half-life of palmitate, DHA, or EPA in brain total phospholipids (Table 3). Similarly, there was no effect of MEP on the t_1/2_ of palmitate, DHA, and EPA in phospholipid fractions except for EPA in ChoGpl where MEP treatment significantly increased the t_1/2_ of EPA by 1.7-fold (Table 3). In addition, MEP did not significantly affect the t_1/2_ of EPA-synthesized n-3 DPA except in PtdSer where there was a significant increase in the t_1/2_ by 1.6-fold (Table 3). Lastly, MEP significantly increased the t_1/2_ of EPA-synthesized DHA in brain total phospholipids, ChoGpl, and EtnGpl by 4.2-, 4.3-, and 2.5-fold, respectively (Table 3). The t_1/2_ of DHA was 4-fold lower than EPA upon vehicle injection (Table 3).

## DISCUSSION

When β-oxidation was inhibited by MEP, we observed significant reductions in radioactivity of the aqueous fractions for ^14^C-palmitate-infused rats by 74%, ^14^C-EPA-infused rats by 54%, and ^14^C-DHA-infused rats by 23% (Figs. 2 and 3). The relatively small reduction in the brain aqueous fraction of ^14^C-DHA-infused rats suggests that β-oxidation products are a small percentage of the radioactivity in the aqueous fraction, and the majority of radioactivity in the aqueous fraction may be attributed to other water-soluble DHA metabolites or glycolipids. We also observed significant increases in the esterification of ^14^C-palmtiate into PtdIns and of ^14^C-EPA into PtdSer; whereas there was no significant change in esterification of ^14^C-DHA into brain phospholipids (Fig. 2). The lack of increase in esterification of DHA into brain phospholipids upon MEP treatment is similar to a previous report with ARA, which is the major n-6 PUFA in rat brain phospholipids (27). While there was no effect of MEP on the esterification of ^14^C-palmitate into cholesteryl esters, there was a significant 2-fold increase in the esterification of ^14^C-palmitate into diacylglycerol [*J*_in_ (vehicle), 106 ± 19 nmol/g/day; MEP, 238 ± 34 nmol/g/day; *P* < 0.01] and a 2-fold increase into triacylglycerol [*J*_in_ (vehicle), 140 ± 23 nmol/g/day; MEP, 276 ± 19 nmol/g/day; *P* < 0.01] (data not shown), which was previously observed by Freed et al. (27). Furthermore, there were significant increases in unesterified FAs known to be extensively β-oxidized upon entry to the brain including palmitate (27, 42), linoleate (22), α-linolenate (23), and EPA (5, 6); whereas the concentration of relatively metabolically stable unesterified PUFAs, including ARA (16) and DHA (15), were unaffected. Previously, using GC-MS, we did not detect unesterified EPA in the brain, but our detection limit was 60 pmol (6). In the current study, again we did not detect EPA by GC-MS (data not shown), but upon more sensitive LC-MS/MS we estimated, for the first time, the brain unesterified EPA pool to be 19 pmol/g brain.

In accordance with previous studies (5, 6), β-oxidation of palmitate, EPA, and DHA was confirmed by the synthesis of radiolabeled cholesterol at 1.4, 1.1, and 0.8 nCi/g brain, respectively (data not shown). Moreover, we observed that within 5 min, EPA was metabolized into longer chain PUFAs such as n-3 DPA and DHA via elongation and desaturation, as well as β-oxidized and resynthesized into palmitate via FA synthase. When β-oxidation was inhibited by MEP, there was an increase in EPA elongation to n-3 DPA, but not DHA. This suggests that without β-oxidation to remove the influx of EPA, the brain compensates by elongating some EPA to n-3 DPA, which emerging evidence suggests may be bioactive in the brain (Fig. 3) (43, 44). Subsequently, the significant increase in total radioactivity of brain total phospholipids with MEP treatment was partly the result of increased esterification of radiolabeled n-3 DPA. However, the brain synthesis of DHA from EPA appears inadequate to maintain the turnover of DHA in brain phospholipids because it would require 768 days to replace phospholipid DHA with DHA-CoA synthesized from EPA; whereas it only required about 1 day to replace phospholipid DHA with intact DHA-CoA.

In addition to the metabolism of EPA via elongation and desaturation, we also observed a 12% reduction in λ (vehicle, 0.12 ± 0.014; MEP, 0.24 ± 0.022; *P* < 0.01) and a 16% reduction in EPA-CoA upon MEP treatment suggesting that inhibition of β-oxidation further reduced the recycling of EPA into brain total phospholipids (Fig. 3). Interestingly, upon MEP treatment, DHA-CoA was also reduced by 26%; whereas linoleoyl-CoA and α-linolenoyl-CoA concentrations increased by 25 and 49%, respectively. Although unclear, these changes in acyl-CoA concentrations may be explained by the selectivity of long chain fatty acyl-CoA synthetases where the inhibition of β-oxidation leads to an influx of unmetabolized linoleate and α-linolenate which competes for long chain fatty acyl-CoA synthetases thereby reducing synthesis of EPA-CoA and DHA-CoA (45, 46).

Previously, we had demonstrated that there were differences between DHA and EPA metabolism in the brain, including β-oxidation (5) and loss kinetics (6), which may partially explain the 250- to 300-fold difference in their brain phospholipid levels. In this study, we further explored additional differences in the metabolism of DHA and EPA to explain large differences in their levels. First, there were no significant differences in the *k*_i_* between DHA and EPA, which recapitulated our previous in situ finding (5). Although there was no difference in the *k*_i_* of DHA and EPA, the net rate of incorporation (*J*_in_) of plasma unesterified DHA into brain phospholipids was 3-fold higher than EPA. Specifically, we found that the most striking difference was in the net rate of incorporation (*J*_FA_) of brain DHA-CoA and EPA-CoA into brain phospholipids. The *J*_FA_ of DHA-CoA into brain phospholipids was 154-fold higher than EPA-CoA (Fig. 3). This implies that the major difference in brain DHA and EPA concentration is not due to uptake from the plasma, but rather from recycling within the brain acyl-CoA pool. This large difference in esterification from the acyl-CoA pool may be attributed to a 36-fold difference in brain acyl-CoA concentrations and a 50-fold difference in λ (DHA, 0.0024 ± 0.0003 vs. EPA, 0.12 ± 0.01; *P* < 0.01). This translated to 75% recycling of DHA per day (t_1/2_ of 22 h) and 21% recycling of EPA per day (t_1/2_ of 3.3 days) in brain phospholipids. The lack of EPA recycling in brain phospholipids may explain the rapid loss of EPA (loss t_1/2_: 5 days or 14% per day) (6) from brain phospholipids as compared with DHA (loss t_1/2_: 33 days or 2% per day) (15). Although not measured in this study, it would be interesting to investigate if EPA released from brain phospholipids is converted to bioactive lipid mediators including E-series resolvins (47, 48).

In comparison to previous reports calculating the *J*_in_ of palmitate (724–822 nmol/g/day) (25, 49, 50), our calculated *J*_in_ of palmitate (2,004 nmol/g/day) for adult rats was 2.4- to 2.8-fold higher. However, this difference appears to be driven by the plasma unesterified palmitate concentration as the *k*_i_* of palmitate in our study was comparable to previous reports (19, 20, 25, 27). As compared with our previously reported *J*_out_ for palmitate (469 nmol/g/day) (6), the *J*_in_ exceeded the *J*_out_, but the *J*_in_ may be an overestimate. Albeit, we only detected radiolabeled palmitate in the brains of ^14^C-palmitate-infused rats; we did not identify the position of radiolabeled carbon. Therefore, it is not possible to differentiate between intact infused ^14^C-palmitate and resynthesized ^14^C-palmitate from recycling of ^14^C in de novo FA synthesis (51–56). Similarly, the *J*_out_ may be underestimated because of extensive palmitate β-oxidation and active resynthesis. Future studies that identify the position of radiolabeled carbons could improve the quantification of *J*_in_ and *J*_out_ for palmitate. The *J*_in_ of DHA (17 nmol/g/day) was 89–91% lower than the reported *J*_in_ of 150–190 nmol/g/day (21, 38). The difference was again driven by a discrepancy in plasma unesterified DHA concentration. There are several possible explanations for this discrepancy including: *1*) different strains of rats; *2*) our rat chow did not contain EPA and DHA, which may decrease circulating unesterified EPA and DHA; and/or *3*) the use of heparin. In this study, as opposed to previous kinetic reports, we did not administer heparin which activates endothelial and hepatic lipoprotein lipase which promotes lipolysis of triacylglycerol into unesterified FAs (57, 58). This highlights the importance of comparing kinetics within the same model strains with the same dietary regimen and under the same experimental conditions. In comparison to the previous reported *J*_out_ for DHA (58–257 nmol/g/day) (15), the *J*_out_ exceeded the *J*_in_ suggesting that multiple plasma pools may be required to maintain DHA levels in brain phospholipids. However, as mentioned previously, differences in experimental conditions may account for the discrepancy; hence, comparison of the *J*_in_ and the *J*_out_ under similar experimental conditions is warranted. In the case of EPA, the calculated *J*_in_ of EPA (5.7 nmol/g/day) accounted for 36% of the *J*_out_ (16 nmol/g/day) (6). This implies that the maintenance of EPA levels in brain phospholipids may require other plasma pools in addition to the unesterified FA pool.

In our previous investigations, we consistently found higher esterification of ^14^C-EPA into PtdIns as compared with other phospholipid classes and DHA (5, 6). This is of interest because PtdIns is a key modulator in several signaling cascades (59) and is a candidate therapeutic target for drugs used to treat bipolar disorder, a disorder that where EPA may be efficacious (60–64). Therefore, an interesting aspect of this study was to examine if EPA in PtdIns would increase upon MEP treatment. Upon inhibition of β-oxidation by MEP, we observed no significant changes in the *J*_in_ for EPA into PtdIns. Furthermore, in contrast to our in situ investigation (5), we found that the *k*_i_* of ^14^C-EPA into PtdIns was significantly lower when compared with ^14^C-DHA. The majority of ^14^C-EPA esterification was into ChoGpl and EtnGpl as opposed to PtdIns. There are two possible explanations for these discrepancies: *1*) the brain concentration of EPA in PtdIns may be tightly regulated acutely in vivo, or *2*) increased esterification of ^14^C-EPA into PtdIns may require phospholipid remodeling that does not occur upon acute intravenous infusion in vivo (6). Therefore, a study that traces the time course of the metabolism and remodeling of EPA containing phospholipids is warranted.

In conclusion, the 250- to 300-fold difference in DHA and EPA brain phospholipid levels may be due to multiple redundant mechanisms including β-oxidation, decreased incorporation from the plasma unesterified FA pool, elongation/desaturation to n-3 DPA, and lower recycling within brain phospholipids (Fig. 3). While β-oxidation may play a role in removing EPA from the brain, this process is not necessary to maintain low levels of EPA because inhibition of β-oxidation can be compensated by increasing EPA elongation/desaturation and reducing EPA recycling.

## Supplementary Material

Supplemental Data
