# Supplementary material for: The low levels of eicosapentaenoic acid in rat brain phospholipids are maintained via multiple redundant mechanisms
Source: J Lipid Res. 2013 Sep;54(9):2410–22. doi: 10.1194/jlr.M038505 (PMC3735939; doi:10.1194/jlr.M038505)
Supplement: Supplemental Data [file supp_54_9_2410__index.html]

The low levels of eicosapentaenoic acid in rat brain phospholipids are maintained via multiple redundant mechanisms — The low levels of eicosapentaenoic acid in rat brain phospholipids are maintained via multiple redundant mechanisms — Supplemental Data 

# The low levels of eicosapentaenoic acid in rat brain phospholipids are maintained via multiple redundant mechanisms

## 

**Files in this Data Supplement:**

- Supplemental Figure 1 - Supplemental Figure 1. EPA-CoA mass spectra.
