# Supplementary material for: The low levels of eicosapentaenoic acid in rat brain phospholipids are maintained via multiple redundant mechanisms
Source: J Lipid Res. 2013 Sep;54(9):2410–22. doi: 10.1194/jlr.M038505 (PMC3735939; doi:10.1194/jlr.M038505)

Supplemental Figure 1. LC-MS/MS profile of palmitoyl-CoA (top), DHA-CoA (top) and EPA-CoA (bottom). (A) For palmitate, the MRM transition is 1006 to 499 m/z. (B) For DHA, the MRM transition is 1078 to 571 m/z. (C) For EPA, there are three MRM transitions: 1052 to 545, 1052 to 428 and 1052 to 136 m/z.

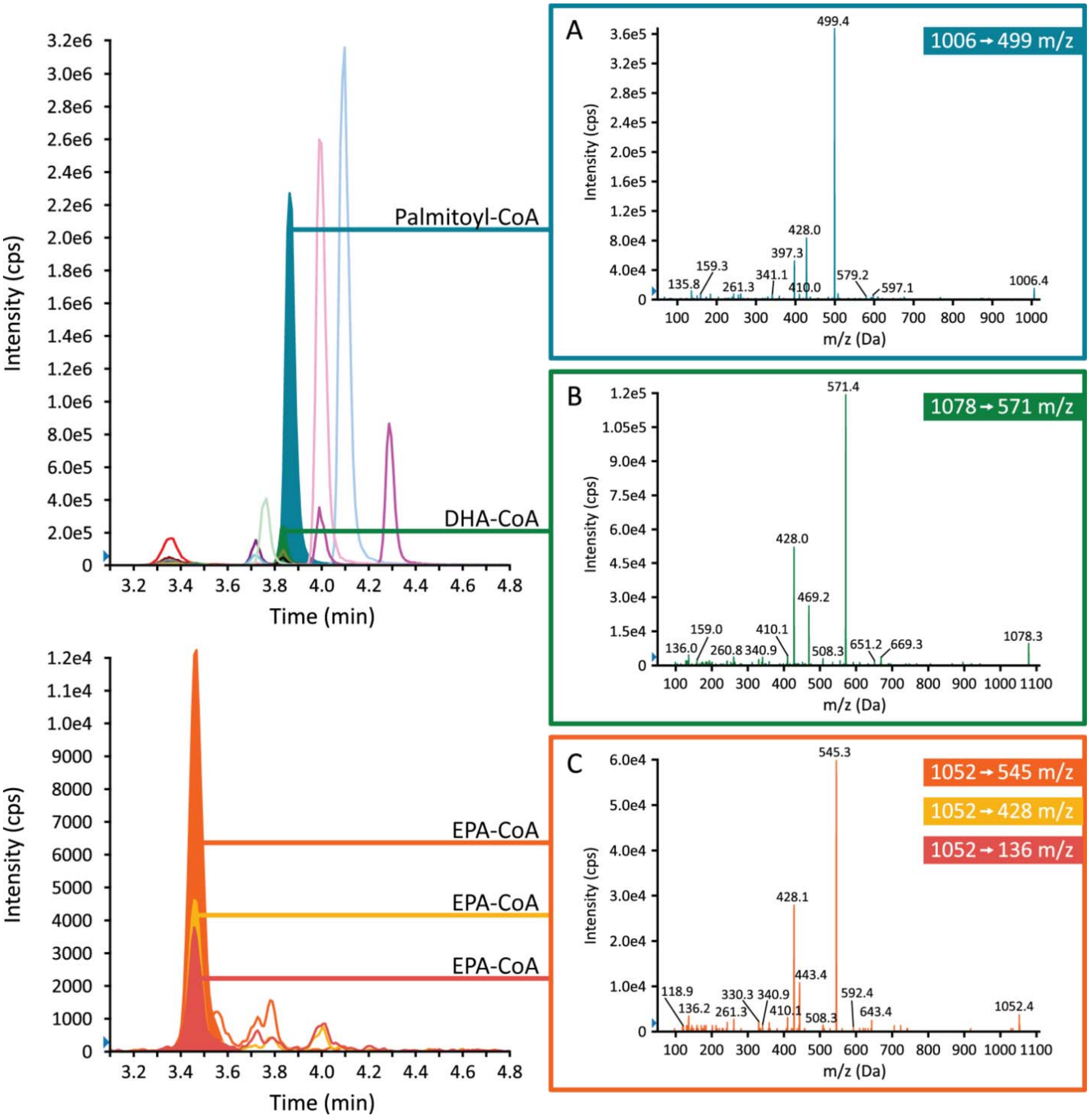

Supplement: Supplemental Data [file supp_M038505_jlr.M038505-1.pdf]
